# Decoding the role of flavonoids in ROS management during heat stress in tomato pollen

**DOI:** 10.1093/plcell/koae234

**Published:** 2024-08-19

**Authors:** Nitin Uttam Kamble

**Affiliations:** The Plant Cell, American Society of Plant Biologists; John Innes Centre, Norwich Research Park, Norwich NR4 7UH, UK

Without the luxury of air conditioning, scorching heat can be brutal for plants. Extreme temperatures adversely affect multiple phases of plant reproduction, particularly pollen viability and germination. A 4 °C to 6 °C increase above optimal temperatures during sexual reproduction negatively impacts crop yields by increasing reactive oxygen species (ROS) levels. While controlled, localized ROS bursts are essential for pollen growth and development, excessive ROS can be detrimental (De Storme and Geelen 2014; Kaya et al. 2014). To counter these damaging effects, plants produce specialized metabolites like flavonoids (flavonols and anthocyanins) with antioxidant properties that protect against harmful ROS. The tomato *anthocyanin reduced* (*are*) mutant, with a mutation *Flavanone 3-Hydroxylase* (*F3H*), accumulates reduced flavonoid levels in its roots, leaves, and pollen compared with wild-type plants (Maloney et al. 2014). The *are* mutant exhibits decreased pollen viability and pollen tube elongation at normal temperatures, which is reversed when the mutant is complemented with the full-length *F3H* gene. The *are* mutant at normal and elevated temperatures shows higher incidence of pollen tube rupture (Muhlemann et al. 2018). Therefore, the *are* mutant serves as an excellent tool to understand the precise mechanisms of heat-induced ROS generation and their oxidative targets, as well as uncovering how high temperatures hinder pollen growth and development at the molecular and cellular levels is crucial to counter reproductive failure.

In new work, **Anthony E. Postiglione and colleagues** (Postiglione et al. 2024) investigated the effect of high temperatures on various aspects of tomato pollen performance in the wild-type cultivar VF36, the *are* mutant, *Pro35S:F3H* overexpression lines in VF36, and complementation lines in the *are* mutant backgrounds. Using the fluorescent chemical probe DPBA (diphenyl-boric acid 2-aminoethyl ester), the authors confirmed that the *F3H* gene increased flavonol levels in the anther tissue and pollen tubes when transformed into VF36 and *are* mutants (see Fig. 1). The hyper-accumulation of flavonols in these lines protected their pollen from high-temperature stress, leading to improved viability, germination rates, tube growth, and overall pollen integrity compared with the *are* mutant (see Fig. 1). The successful genetic complementation mitigated the reduction in pollen viability and thermotolerance in the *are* mutant, restoring it to wild-type VF36-like levels.

**Figure 1.**
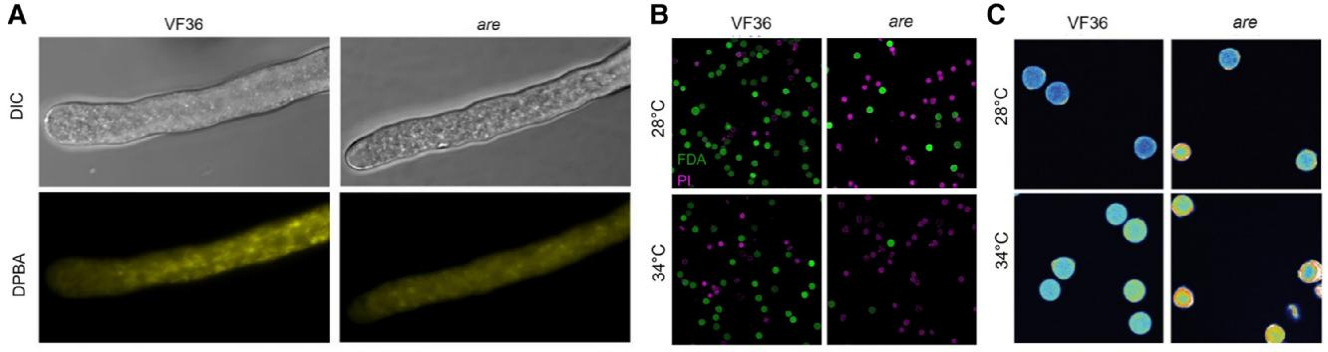
Flavonols improve tomato pollen thermotolerance. **A)** Flavonol levels in pollen tubes of VF36 and the *are* mutant as visualized by DPBA (diphenyl-boric acid 2-aminoethyl ester) staining. **B)** Pollen viability, assessed using FDA and propidium iodide staining (green, live grains; magenta, dead grains). **C)** ROS accumulation in the germinating pollen grain of VF36 and *are* mutant, analyzed using CM-H2DCFDA staining. Reprinted from Postiglione et al. (2024) Figs. 3A; 2, B and 6A.

Postiglione et al. observed similar results using chemical complementation, where exogenous application of the flavonol kaempferol enhanced heat tolerance and reduced temperature hypersensitivity in the *are* mutant pollen. The authors also noted that pollen flavonols enhanced pollen tube growth through the stigma upon fertilization. They further observed that at both low and high temperatures, the *are* mutant pollen showed enhanced ROS accumulation as visualized using the CM-H2DCFDA and PO1 chemical probe compared with the wild-type VF36 line, which was reversed by the *Pro35S:F3H* gene (see Fig. 1). These findings indicate that genetic and chemical complementation of flavonols effectively reduced the impact of the *are* mutation on ROS accumulation and improved pollen performance.

To gain insight into the role of ROS production during temperature stress, the authors explored the potential of inhibiting enzymatic mediators of ROS synthesis. The Respiratory Burst Oxidase Homologs (RBOH) inhibitor VAS2870 significantly reduced the negative impact of high temperatures on pollen tube lengths, confirming that RBOHs contribute to excessive ROS accumulation under heat stress and that the *are* genotype's performance could be improved using RBOH inhibitors.

Postiglione et al. explored the transcriptional regulation during heat stress using transcriptome analysis in VF36, the *are* mutant, and *Pro35S:F3H* overexpression lines in VF36 under both optimal and elevated temperature conditions. Their analysis suggests that at elevated temperatures, the *are* mutant has a greater number of unique transcripts that are differentially expressed with higher abundance compared with other genotypes. The authors also observed that *F3H* expression was elevated in *are* compared with VF36, suggesting that a flavonol intermediate may act as a negative feedback signal. They also compared the core set of temperature-dependent DEGs, including heat shock proteins, heat shock transcription factors, and molecular chaperones in *are* and VF36, noting that most of the genes were upregulated in *are* even at optimal temperatures. However, their transcriptomic analysis could not completely explain the impaired performance of *are* pollen at optimal temperatures.

In conclusion, this study offers valuable insights into the role of flavonoids in response to heat stress. It also highlights the role of RBOH enzymes in ROS synthesis and reveals a strong transcriptional response to heat, which is intensified by low flavonol levels in pollen grains and pollen tubes. Overall, flavonoids are vital for the ability of plant male reproductive tissues to cope with heat stress and could be a promising target for engineering crops with improved temperature tolerance. Crop breeding strategies that specifically aim to protect reproductive tissues from temperature-induced ROS stress while improving plant performance will especially benefit from the findings of this study.

## Dive Curated Terms

The following phenotypic, genotypic, and functional terms are of significance to the work described in this paper:

F3H Gramene: AT3G51240
F3H Araport: AT3G51240

flavonols CHEBI: CHEBI:28802
